# Energy intake and energy expenditure of pre-professional female contemporary dancers

**DOI:** 10.1371/journal.pone.0171998

**Published:** 2017-02-17

**Authors:** Meghan A. Brown, Glyn Howatson, Edel Quin, Emma Redding, Emma J. Stevenson

**Affiliations:** 1 Faculty of Health and Life Sciences, Northumbria University, Newcastle upon Tyne, NE1 8ST, United Kingdom; 2 School of Sport and Exercise, University of Gloucestershire, Gloucester, GL2 9HW, United Kingdom; 3 Water Research Group, School of Biological Sciences, North West University, Potchefstroom, South Africa; 4 Faculty of Dance, Trinity Laban Conservatoire of Music and Dance, London, SE8 3DZ, United Kingdom; 5 Institute of Cellular Medicine, Newcastle University, Newcastle, NE2 4HH, United Kingdom; University of Texas Southwestern Medical Center, UNITED STATES

## Abstract

Many athletes in aesthetic and weight dependent sports are at risk of energy imbalance. However little is known about the exercise and eating behaviours of highly trained dance populations. This investigation sought to determine the energy intake and energy expenditure of pre-professional female contemporary dancers. Twenty-five female contemporary dance students completed the study. Over a 7-day period, including five week days (with scheduled dance training at a conservatoire) and two weekend days (with no scheduled dance training at the conservatoire), energy intake (self-reported weighed food diary and 24 h dietary recall) and expenditure (tri-axial accelerometry) were recorded. Mean daily energy intake and expenditure were different over the 7-day period (*P* = 0.014) equating to an energy deficit of -356 ± 668 kcal·day^-1^ (or -1.5 ± 2.8 MJ·day^-1^). Energy expenditure was not different when comparing week and weekend days (*P* = 0.297). However daily energy intake (*P* = 0.002), energy availability (*P* = 0.003), and energy balance (*P* = 0.004) were lower during the week compared to the weekend, where energy balance became positive. The percentage contribution of macronutrients to total energy intake also differed; with higher fat (*P* = 0.022) and alcohol (*P* = 0.020), and lower carbohydrate (*P* = 0.001) and a trend for lower protein (*P* = 0.051) at the weekend. Energy balance and appropriate macronutrient intake are essential for maintaining the demands of training, performance and recovery. Whilst aesthetics are important, female contemporary dancers may be at risk of the numerous health and performance impairments associated with negative energy balance, particularly during periods of scheduled training.

## Introduction

Dance is characterised as a moderate-high intensity, high skill, and predominantly intermittent activity [1]. These characteristics can vary, largely dependent on the style of dance and the capacity in which it is performed. The daily training schedule of a dancer is difficult to define but typically includes multiple training sessions, consisting of technique classes, rehearsals, and/or performances. The intensity and volume of exercise previously reported [2] can often be comparable to that of many elite athletes. In addition, since dance is principally an art form, it demands artistry and expression as well as physical and technical skill. As with many comparable aesthetic sports, while extremely low body mass and fat mass are known to negatively influence performance potential, low levels are nevertheless often considered to be advantageous for movement efficacy and artistic expression [3]. Indeed, maintaining a lean physique is thought to be an important aspect of dance fitness and a pre-requisite for success in the profession [4, 5]. As a result, a dichotomous issue arises in dance, whereby attaining the desired body composition can be a conflicting component in the pursuit of optimal performance.

The typical energy intake and energy expenditure of athletes has been explored in a number of sports, for instance in football [6, 7], taekwondo [8], and gymnastics [9]. However little is known about the nutritional intake and energy requirements of a dance population. This is surprising given that many athletes in aesthetic or weight dependent sports fail to compensate high energy demands with an adequate energy intake, and are at risk of numerous health and performance impairments associated with energy imbalance [10]. A recent review [1] has summarised the research investigating the energy demands of dance; largely through measurement of heart rate and oxygen cost. Though the authors conclude that the majority of investigations describe the energy demand to be moderate-high and intermittent, they noted a number of methodological limitations. Additionally, while these studies have identified energy demands in regards to a single movement, class, or performance, few have investigated these in nutritional contexts (i.e. kcal) or investigated the longer-term energy demands. Similarly, while a number of studies have sought to identify the dietary intakes of dancers, only a handful have looked at this in parallel with their physical activity or energy expenditure. The majority of these studies determined that dancers were (for the most part) in negative energy balance or very low energy availability [11–19]. However, these investigations used a range of measurement techniques to estimate energy intake and energy expenditure, and whilst these techniques provide an indication of energy balance, their validity has been questioned; thus limiting the strength of their findings. For instance, heart rate monitoring is not a reliable indicator for estimations of energy demand given the intermittent nature of dance [20], and self-reported dietary records are limited by under/over eating and/or reporting [21]. Indeed, a study in female ballet dancers reported a mean bias to under-reporting of 667 kcal·day^-1^ or 21% of energy intake when comparing four-day weighed food recording and energy expenditure via doubly labelled water [22].

Although the low body mass index and body fat levels frequently reported in dancers [23–25] suggest that exercise and/or eating behaviours may be suboptimal, the inherent limitations in study designs render previous conclusions of poor nutritional intake and energy balance questionable. Moreover, the majority of research has been conducted in ballet and little has been published in modern/contemporary equivalents. Research has demonstrated differences in artistic and physical demands of ballet and contemporary styles [26] and body composition data reveal that ballet dancers tend to be the leanest [27]. Thus it is not appropriate to assume that the literature regarding one dance style is relevant and directly transferable to the other.

Whilst aesthetics are important, dancers are subject to similar physiological stressors as other athletes, and dietary habits can affect dance performance [28]. Consequently, dancers would benefit from a greater understanding of their energy requirements to support their training schedules. Therefore this investigation sought to determine the energy and macronutrient intake and energy expenditure of pre-professional female contemporary dancers during a 7-day period of full time training at a conservatoire. A secondary objective was to compare exercise and dietary behaviours during week days (Monday-Friday; where there was scheduled dance training), and during the weekend (Saturday and Sunday; where there was no scheduled dance training).

## Materials and methods

### Participants

Twenty-five pre-professional female undergraduate contemporary dance students attending a conservatoire volunteered for the study (mean ± SD age 21 ± 2 y; stature 167.4 ± 5.9 cm; mass 63.4 ± 6.9 kg; and BMI 22.6 ± 2.0 kg·m^2^). Prior to recruitment, institutional ethical approval was granted by the Faculty of Health and Life Sciences Ethics Committee of Northumbria University in Newcastle upon Tyne, UK, and written informed consent obtained. Participants were free from injury and currently participating fully in all scheduled dance training (*n* = 14, *n* = 8 and *n* = 3 in 1^st^, 2^nd^ and 3^rd^ years, respectively of a three-year full-time undergraduate dance degree). All participants were instructed to maintain their typical dietary and physical activity behaviours throughout the study.

#### Questionnaires

Participants completed the Healthier Dance Practice National Survey (also referred to as the Fit to Dance 2 national survey) [24] to provide a range of information including dance background as well as dietary history. A menstrual cycle questionnaire was completed to assess menstrual cycle history and, where possible, to identify menstrual cycle phase. In addition an 18 item, Three Factor, Eating Questionnaire (TFEQ-R18) [29] was used to assess three eating behaviours; restrained eating (conscious restriction of food intake in order to control body weight or to promote weight loss), uncontrolled eating (tendency to eat more than usual due to a loss of control over intake accompanied by subjective feelings of hunger), and emotional eating (inability to resist emotional cues). The degree of expression (0–100%) of each eating behaviour was determined by comparing absolute scores relative to the proportion of the highest possible scores; with higher values indicating more of the behaviour [30].

### Body composition

Stature was measured to the nearest 1 mm (Seca, Birmingham, UK) and body mass to the nearest 0.1 kg (Seca, Birmingham, UK). As per the standard techniques of the International Society for the Advancement of Kinanthropometry (ISAK) [31], calibrated Harpenden calipers (CMS Weighing Equipment Ltd, London, UK) were used for skinfold measurement at 7 sites (biceps, triceps, subscapular, suprailiac, abdominal, front thigh and medial calf) and waist and hip circumference was measured to determine waist to hip ratio (W:H). All measurements were taken on the right side of the body and measured in duplicate and the mean of these was used for analysis. Participants were fasted for ≥ 2 hours prior to measurement and all measurements were taken by the same investigator who demonstrated good intra-rater reliability (TEM 4% and 2% for skinfold and circumference respectively). Percentage body fat (%BF) was estimated [32] from predicted body density [33]. This then allowed for estimation of fat and fat free mass (FFM).

### Energy intake

Participants were asked to complete a 7-day weighed food diary to provide a detailed description of their food and fluid intake as well as supplement use if applicable. Participants were given comprehensive verbal and written instructions to report time of consumption, how food/fluid was cooked or prepared, brand names, and quantities; electronic portable scales were provided.

In addition each participant engaged in a 24-hour recall interview using the two-pass method [34] on each day of the data collection period to be cross-referenced with the food diary. This allowed the researcher to clarify ambiguous information and complete diary entries with missing data. Commercially available dietary analysis software (Nutritics Ltd, Ireland) was used to calculate total energy intake (TEI). A single researcher analysed the dietary data in order to avoid variability in interpretation of these data [35]. Where foods were not listed in the dietary database, the product label was consulted and the nutrient composition entered manually. All testing was conducted in free living conditions and no attempts were made to influence the diet of participants.

### Energy expenditure

A tri-axial accelerometer (ActiGraph GT3X+, Florida, USA) was secured under clothing on the right hip. These were worn continuously (except during activities which would submerge the accelerometer in water) throughout the same 7-day period that was analysed for dietary intake. Sixty second sampling epochs were collected at a 30 Hz sample rate. The Freedson VM3 combination algorithm [36] was used to estimate physical activity energy expenditure from the vector magnitude counts per minute of the three axis.

While participants were asked to wear the accelerometer at all times (except for washing and swimming), accelerometers were removed periodically for legitimate reasons; for example discomfort during sleep or when it was prohibited during performances. To account for missing data during these times, participants were required to register all non-wear periods (the activity, time and duration) in a diary [37]. Appropriate Metabolic Equivalent (MET) values from the Compendium of Physical Activities [38] were assigned to these reported activities, and were subsequently corrected to account for individual variation [39]. Corrected METs were used to estimate non-wear energy expenditure [38].

Individual basal metabolic rate (BMR) [40] was also estimated as well as the thermic effect of food (TEF). The TEF varies among macronutrients and as applied in previous research [6], the following values were used to calculate the total TEF in the present study; 2.5, 7, and 27.5% of intake for lipids, carbohydrate and protein, respectively.

### Statistical analysis

The physical activity energy expenditure estimated from the accelerometer and from non-wear activity, BMR, and TEF were combined to estimate individual total energy expenditure (TEE). The average TEE (kcal, MJ), TEI (kcal, MJ), energy balance (kcal, MJ), energy availability (kcal·kg FFM), and macronutrient contributions (% of TEI, g and g·kg^-1^) were determined for three periods; the total 7-day data collection period, an average day of the week (scheduled dance training), and an average weekend day (no scheduled dance training). Three separate paired sample *t*-tests were used to compare energy intake and energy expenditure during each of the three periods (7-day period, day of the week, and weekend day) in order to assess energy balance. Paired sample *t*-tests were also conducted on all variables to compare an average week day and an average weekend day. Unless otherwise stated, 95% confidence intervals (95% CI) are presented for mean differences between an average week day and an average weekend day. Statistical software (IBM SPSS v22, IBM, USA) was used and significance accepted at the *P* ≤ 0.05 *a priori*.

## Results

### Participant demographics

Participant characteristics are presented in Table 1. The Healthier Dance Practice National Survey determined that 7 participants were either vegan, vegetarian or actively avoided red meat, 6 reported to not drink alcohol, 2 were following weight-reducing diets, and 9 reported past and/or current eating problems. Despite this, participants demonstrated average levels of cognitive restraint (49 ± 20%), uncontrolled eating (44 ± 14%), and emotional eating (47 ± 22%), as evidenced by the TFEQ-R18. Self-reported menstrual function determined that 14 were eumenorrheic, 9 were oligomenorrheic, and 2 could not be determined due to contraceptive hormone use. Contraceptive use varied; 16 were naturally menstruating, 6 were using combination pill/vaginal ring/contraceptive patch, 2 were using progesterone only pills/implant, 1 used an oestrogen replacement. There was difficulty in identifying the menstrual cycle phase in 7 participants (due to irregular cycles or particular contraceptive hormone use) and were therefore undetermined, while 9 were in the follicular phase, 4 in the luteal phase, and 5 in late luteal and early follicular phases during the 7-day data collection period.

**Table 1:** Participant characteristics, *n* = 25, mean ± SD.

Age (y)	21 ± 2
Body mass (kg)	63.4 ± 6.9
Stature (cm)	167.4 ± 5.9
BMI (kg·m^2^)	22.6 ± 2.0
Waist: Hip	0.74 ± 0.03
Body fat (%)	28.0 ± 3.4
Fat free mass (kg)	45.5 ± 4.3
Self-reported physical activity (h·week^-1^)	26.3 ± 5.8
Dance training ≥ 10 h·week^-1^ (y)	5 ± 3

### Energy intake and energy expenditure

**Fig 1:**
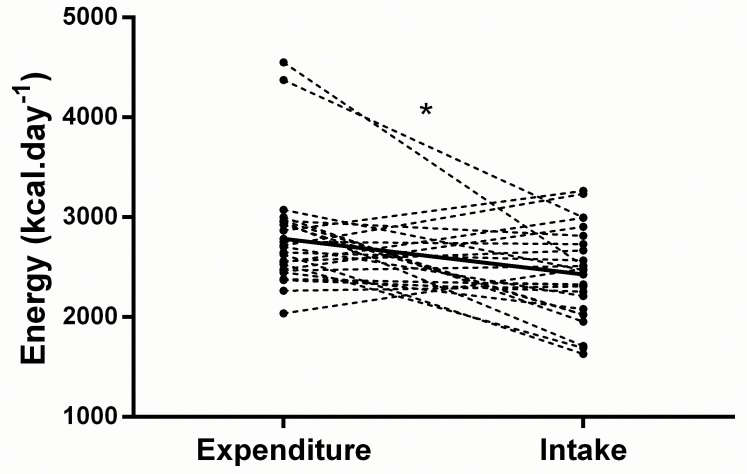
Energy intake and energy expenditure for each individual (*n* = 25) over the 7-day data collection period, and the group mean (dashed line). *denotes group mean significant difference (*P* < 0.05).

**Fig 2:**
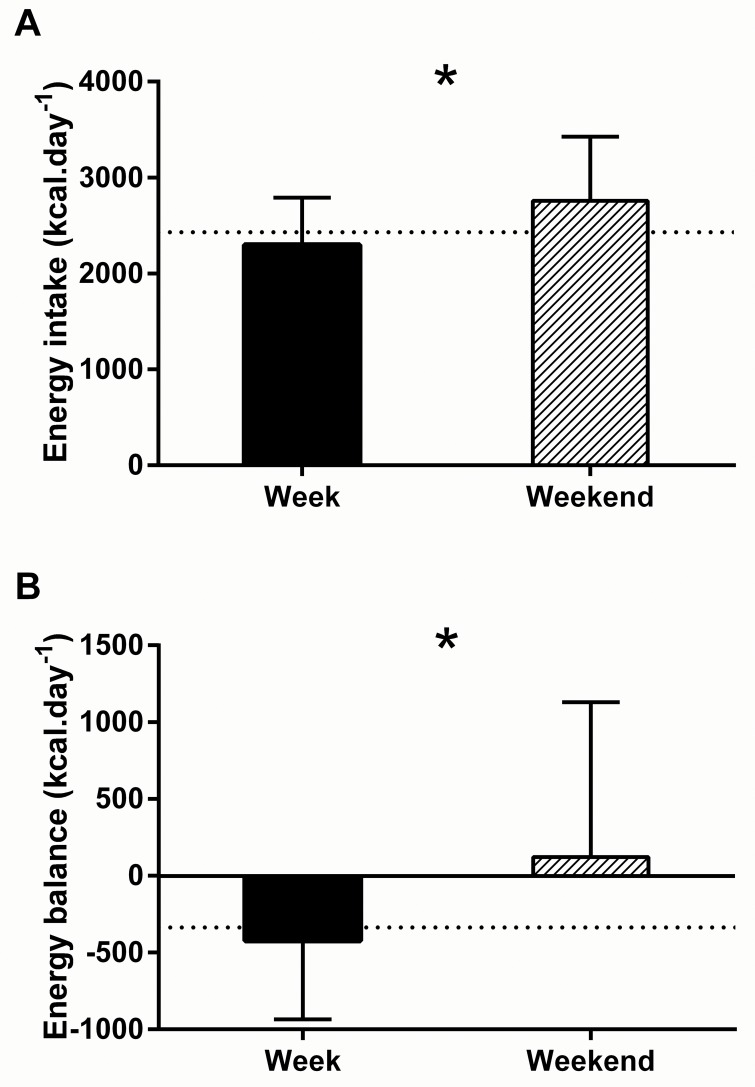
**Energy intake (A) and energy balance (B) of participants (*n* = 25) during an average week day, and an average weekend day.** The dashed line represents group mean over the total 7-day data collection period. Values presented as mean ± SD. *denotes group mean significant difference between week and weekend (*P* < 0.05).

**Fig 3:**
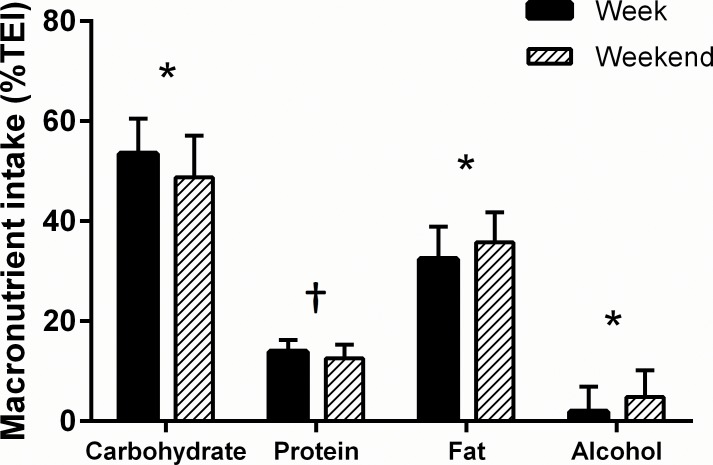
Percentage contributions to total energy intake (% TEI) of carbohydrate, protein fat, and alcohol during an average week day and an average weekend day. Values presented as mean ± SD. *denotes group mean significant difference (*P* < 0.05) and ^†^denotes a trend (*P* = 0.051) towards significant difference between week and weekend.

Total energy expenditure, and energy and macronutrient intakes are summarised in Table 2. Fig 1 illustrates the daily energy intake and expenditure of each individual across the 7-day period. Average energy intake was lower than energy expenditure during the 7-day period (2428 ± 458 kcal or 10.2 ± 1.9 MJ vs 2784 ± 569 kcal or 11.6 ± 2.4 MJ, *P* = 0.014, [95% CI: -632, -80]) equating to an energy deficit of -356 ± 668 kcal·day^-1^ or -1.5 ± 2.8 MJ·day^-1^. Energy expenditure did not differ when comparing week and weekend days (2719 ± 407 vs 2633 ± 574 kcal, *P* = 0.297, [95% CI: -80, 252]). However daily energy intake (2297 ± 492 vs 2756 ± 669 kcal, *P* = 0.002, [95% CI: -735, -183]) (Fig 2A), energy availability (24 ± 10 vs 36 ± 21, kcal·kg FFM^-1^, *P* = 0.003, [95% CI: -20, -4]), and energy balance (-422± 513 vs 123 ± 1007 kcal *P* = 0.004, [95% CI: -902, -187]) (Fig 2B) were lower during the week (during scheduled dance training) compared to the weekend, where energy balance in fact became positive. Percentage contributions to TEI of carbohydrate, fat and alcohol differed between an average week day and an average weekend day; where carbohydrate was lower (*P* = 0.001, [95% CI: -7, -2%]) but alcohol and fat intake was highest at the weekend (*P* = 0.022, [95% CI: 1, 6%]; and *P* = 0.020 [95% CI: 1, 5%] respectively). There was a strong trend for a lower percentage of TEI derived from protein at the weekend (*P* = 0.051, [95% CI: -1, 3%]) as illustrated in Fig 3.

**Table 2:** Energy expenditure and energy and macronutrient intakes, *n* = 25, mean ± SD.

Variable		7-day	Week	Weekend
**Energy**				
Expenditure	kcal·day^-1^	2784 ± 569	2719 ± 407	2633 ± 574
	MJ·day^-1^	11.6 ± 2.4	11.4 ± 1.7	11.0 ± 2.4
Intake	kcal·day^-1^	2428 ± 458	2297 ± 492*	2756 ± 669*
	MJ·day^-1^	10.2 ± 1.9	9.6 ± 2.1*	11.5 ± 2.8*
Balance	kcal·day^-1^	-356 ± 668	-422 ± 513*	123 ± 1007*
	MJ·day^-1^	-1.5 ± 2.8	-1.8 ± 2.1*	0.5 ± 4.2*
Availability	kcal·kg FFM^-1^·day^-1^	26 ± 13	24 ± 10*	36 ± 21*
**Carbohydrate**	g·day^-1^	313 ± 58	304 ± 57	335 ± 97
	g.kg^-1^·day^-1^	5.0 ± 1.0	4.8 ± 0.8	5.4 ± 1.7
	% of total energy intake	52 ± 7	54 ± 7*	49 ± 8*
**Protein**	g·day^-1^	81 ± 15	79 ± 17	85 ± 22
	g.kg^-1^·day^-1^	1.3 ± 0.3	1.3 ± 0.3	1.4 ± 0.5
	% of total energy intake	13 ± 2	14 ± 2^†^	13 ± 3^†^
**Fat**	g·day^-1^	92 ± 30	85 ± 33*	110 ± 33*
	g.kg^-1^·day^-1^	1.5 ± 0.4	1.3 ± 0.5*	1.8 ± 0.6*
	% of total energy intake	34 ± 5	32 ± 6*	36 ± 6*
**Alcohol**	g·day^-1^	9 ± 13	5 ± 14*	20 ± 22*
	g.kg^-1^·day^-1^	0.2 ± 0.2	0.1 ± 0.3*	0.3 ± 0.4*
	% of total energy intake	3 ± 4	2 ± 5*	5 ± 5*

*P < 0.05

^†^*P* = 0.051: difference between week and weekend.

## Discussion

This study aimed to investigate the energy intake and expenditure of female contemporary dancers during a week of full-time, pre-professional dance training. The present investigation was the first to utilise accelerometry and the combined method of self-report weighed food diaries and dietary recall interview techniques. In agreement with the literature [11–19], we found that on average dancers were in negative energy balance (17 of 25 participants) with a daily deficit of -356 ± 668 kcal (-1.5 ± 2.8 MJ).

While the average energy deficit observed in the present study is less than previously reported in some dance populations (recently a deficit of -2.35 ± 2.14 MJ·day^-1^ in female ballet dancers [13]), chronic energy deficiency in athletes can lead to detriments in performance and subsequent recovery, and could compromise growth, maturation and health [10]. For instance potential issues arising from inadequate nutrition in dancers include insufficient peak bone mass and menstrual dysfunction [19, 41]. Female dancers (as with other athletic females) are recommended to maintain an energy availability above 30 kcal·kg FFM^-1^·day^-1^ to reduce the risk of disorders associated with energy imbalance [42]; the present study demonstrated this to be only 26 ± 13 kcal·kg FFM^-1^·day^-1^. Accordingly, contemporary dancers would benefit from a greater understanding of their energy requirements and certainly more education regarding appropriate nutritional strategies to support their training demands.

Carbohydrates play an essential role in exercise metabolism and the delay of fatigue, as well as contributing to the replenishment of glycogen stores during recovery. We observed intakes of 5.0 ± 1.0 g·kg^-1^·day^-1^ over the 7-day period, which narrowly achieve the 5–7 g·kg^-1^·day^-1^ recommended for athletic populations [43], and the average 4.8 g·kg^-1^·day^-1^ during the academic training week falls short of these guidelines. Given that much of contemporary training is typically of low-moderate intensity [26] conducive to beta-oxidation, this is likely sufficient. However during more high-intensity training and/or performance periods, dancers may consider increasing carbohydrate intake in order to meet the higher physiological demands [1]. Similarly, adequate protein intake and amino acid availability are necessary for the repair and remodelling of skeletal muscle and connective tissue after exercise [44], which is critical given that dance has been shown to induce muscle damage [45]. The average intakes of 1.3 ± 0.3 g·kg^-1^·day^-1^ in the current investigation meet recommendations of 1.2–1.7 g·kg^-1^·day^-1^ [46]. However, recent research demonstrates that muscle protein synthesis is down-regulated when in energy deficiency and as a result energy deficient individuals should consume high protein diets (1.6–2.4 g·kg^-1^·day^-1^) to restore muscle protein synthesis and attenuate proteolysis and skeletal muscle loss [47, 48]. This evidence suggests that intakes in this population are likely below optimal, particularly given that 7 of the 25 participants were vegan, vegetarian or actively avoided red meat. Therefore the protein sources they consume may be of predominantly low biological value, for instance in vegetables, legumes, nuts, and grains. While fat plays a role in many physiological processes, fat intake represented 34 ± 5% of TEI (1.5 ± 0.4 g·kg^-1^·day^-1^) and was higher still at the weekend (1.8 ± 0.1 g·kg^-1^·day^-1^; representing 36 ± 6% of TEI); above recommended levels of <30%. Similarly, alcohol intakes were relatively high, contributing 3 ± 4% and 5 ± 5% of TEI across the 7-day period and on an average weekend day respectively. This is concerning given the evidence which suggests that alcohol intake is associated with reduced muscle protein synthesis [49], impaired glycogen restoration [50], and exacerbated losses in muscle function [51]. Certainly the dancers would benefit from a reduction in alcohol intake, specifically limiting intake to 0.5 g·kg^-1^ in any post-exercise period in order to avoid interference with recovery processes [52].

Interestingly, the eating behaviours of these dancers are somewhat different during scheduled dance training (during the week) compared to when there is no scheduled dance training at the conservatoire (during the weekend). While we have demonstrated energy deficits throughout the week, the maintenance of similar energy demands (as a result of many of the dancers seeking extra-curricular classes and/or training) and increased energy intake contributed to a positive energy balance during the weekend (123 ± 1007 kcal·day^-1^ or 0.5 ± 4.2 MJ·day^-1^). Perhaps the dancers perceived that while they were not in training they could indulge in arguably less desirable nutritional behaviours. In contrast, during periods of academic dance training the participants appeared to respond with below optimal energy and macronutrient intakes for their training demands. Though restrictive, uncontrolled, and emotional eating behaviours appeared not to be elevated, a relatively high proportion of participants reported past and/or current eating problems (9 of 25 participants), which may be responsible for the erratic eating behaviours. In addition, the typical training schedule of a dancer offers unpredictable and/or limited opportunities for food and drink consumption, likely exacerbating these issues. Indeed an early investigation suggested that binge eating, particularly at the weekend, might explain why dancers do not experience reductions in weight [53]. Moreover, it is possible that the fluctuations in energy balance and macronutrient contributions we have observed from day-to-day (week day/weekend) are occurring in the long term (term time/off-season). This may explain why the dancers’ body composition reported in the present investigation were healthy, despite an average negative energy balance indicative of weight loss. Indeed, the body mass index and percentage body fat of the participants were higher than typically reported in ballet populations (for instance 18.9 ± 1.0 kg·m^2^ and 17.4 ± 3.4% respectively in female ballet dancers [25]). However, given the differences in physiological demands as well as discrete skills between these dance genres (not least the gender roles in ballet requiring females to be lifted more frequently) [26], this is perhaps unsurprising. Collectively, these data suggest that the dancers are unable to effectively regulate their energy and macronutrient intakes to accommodate for their energy expenditure; essential for maintaining the demands of training, performance, recovery and physiological adaptation.

Accurately quantifying energy intake and energy expenditure is limited by indirect measurement techniques typically relied upon in research studies. In the present study, a 7-day period was chosen as it is thought to best represent the variety of dietary and physical activity practices and are associated with the most valid nutritional information [54]. However, it has been suggested that reasons for low energy intakes reported by dietary records in dance populations include deliberate under-reporting and/or under-eating due to the desire for weight loss [11, 18]. As observed in many populations, it is thought that not only is it possible that dancers fail to record portions of food correctly, but they may also omit foods eaten or restrict their food intake during the study period [12]. This should be considered when interpreting the present findings. However, in order to minimise the errors of measurement associated with participant compliance and motivation, we combined self-reported weighed food diaries with 24-hour recall interviews. This method has been found to result in good agreement with the gold standard observed food intake technique [55, 56]. Moreover, though accelerometry has been shown to be strongly correlated with indirect calorimetry [57, 58], some show an underestimation of physical activity levels using the devices [59–61]. Notwithstanding, the loss of data associated with removal of the accelerometer evidently influences the estimation of physical activity [62]. Though efforts were made to account for missing data using non-wear activity logs, the accuracy of these is nevertheless limited by the possibility that 1) not all activities were reported and accounted for, and 2) the information provided was lacking detail. It is also important to note that demanding exercise and energy expenditure may alter BMR; particularly during energy deficit. Future research should consider more accurate estimation of BMR to determine its contribution to TEE. Finally, energy balance may differ across the menstrual cycle, for instance energy intake and expenditure appear to be elevated during the luteal phase [63]. In the present investigation, the menstrual cycle phase of participants during the study differed and in some cases was not identifiable, and therefore the findings should be interpreted with this in mind. Despite the aforementioned limitations, the present study offers a high degree of ecological validity in its free-living experimental design, which may not have been possible with other experimental techniques.

## Conclusions

To conclude this research demonstrates that, as with many athletes in aesthetic or weight dependent sports, female contemporary dancers are at risk of energy deficiency, particularly during periods of scheduled dance training. As a result, this population could be susceptible to the numerous health and performance impairments associated with energy imbalance. In addition appropriate macronutrient intakes are essential for maintaining the demands of training, performance and recovery. The suboptimal macronutrient intakes observed in this study suggest a lack of knowledge regarding appropriate nutrition for sport and exercise performance. It is evident that dance populations would benefit from further research in order to develop current understanding of dance specific nutrition. For instance the variability of nutritional intakes and energy expenditures between term time and off-season remain to be investigated, and whether the negative energy balance reported in the current study is maintained in the long-term. Moreover it may be prudent to investigate nutritional strategies and the use of ergogenic aids which may provide practical solutions to improve energy balance and perhaps contribute to enhancing performance and recovery.

## Supporting information

S1 Dataset Relevant raw data underlying the study findings. Participant characteristics, energy and macronutrient intakes, energy expenditure, energy balance and energy availability.
(XLSX)
